# Industrial Fungal Enzymes: An Occupational Allergen Perspective

**DOI:** 10.1155/2011/682574

**Published:** 2011-06-21

**Authors:** Brett J. Green, Donald H. Beezhold

**Affiliations:** Allergy and Clinical Immunology Branch, Health Effects Laboratory Division, National Institute for Occupational Safety and Health, Centers for Disease Control and Prevention, Morgantown, WV 26505-2888, USA

## Abstract

Occupational exposure to high-molecular-weight allergens is a risk factor for the development and pathogenesis of IgE-mediated respiratory disease. In some occupational environments, workers are at an increased risk of exposure to fungal enzymes used in industrial production. Fungal enzymes have been associated with adverse health effects in the work place, in particular in baking occupations. Exposure-response relationships have been demonstrated, and atopic workers directly handling fungal enzymes are at an increased risk for IgE-mediated disease and occupational asthma. The utilization of new and emerging fungal enzymes in industrial production will present new occupational exposures. The production of antibody-based immunoassays is necessary for the assessment of occupational exposure and the development of threshold limit values. Allergen avoidance strategies including personal protective equipment, engineering controls, protein encapsulation, and reduction of airborne enzyme concentrations are required to mitigate occupational exposure to fungal enzymes.

## 1. Introduction

In the United States, the 2010 civilian workforce accounted for 139 million people [1] who spend up to a quarter of their lifetime and half of their waking lives at work [2]. With changes in the global market, particularly with the rise of biotechnology, new occupational hazards have emerged. Approximately, 200 biotic (organisms or particles of viral, prokaryote, or eukaryote origin) and an even greater number of abiotic (physical and chemical) agents have been associated with adverse health outcomes. In certain occupational settings, particularly those engaged in handling purified microbial proteins in baking and manufacturing sectors, workers are at increased risk of becoming sensitized and developing respiratory disease.

Occupational asthma (OA) is the most common respiratory disease reported in the workplace [3–8]. OA has been defined as either irritant induced or immune mediated [6, 7]. Immunologically mediated OA accounts for approximately 90% of cases [9], but the severity of disease is dependent on the concentration, route, agent of exposure, and the latency period [6, 7]. Both high- and low-molecular-weight antigens can induce OA, but the immunological mechanisms are distinctly different. High-molecular-weight allergens are generally proteins that are greater than 5 kDa, and production of immunoglobulin E (IgE) results in the release of mediators from mast cells and eosinophils [6, 7].

More than 250 high-molecular-weight allergens that induce OA have been identified [4, 6, 7]. Many are derived from animals or plants, and exposure usually involves mixtures of many proteins [4, 6]. Occupations where high-molecular-weight allergens have been characterized include seafood processing (tropomysin), dairy, poultry, citrus, greenhouse, baking, healthcare (latex), pharmaceutical (drugs), and detergent manufacturing (fungal enzymes) [6]. Some of the best examples of high-molecular-weight occupational allergens are the fungal enzymes. They are particularly suited for study because they are often used as purified preparations in baking, food, detergent, textile, and pharmaceutical industries [6, 10]. In this paper, we will focus on the fungal enzymes as model high-molecular-weight allergens in industrial settings and describe the main enzymes that have been associated with occupational sensitization and asthma. Identification of emerging fungal enzymes in manufacturing and biotechnology industries is discussed as well as new methods to detect and quantify fungal enzymes in the occupational environment. Methods to avoid fungal allergens in the workplace are additionally covered. 

## 2. Fungal Enzymes in Occupational Disease

The industrial utility of fungi has been well known since antiquity. In addition to the role of fungi as saprophytes in the environment, many species have commercial use, for example, mushrooms as food sources, ingredients in food preparation (cheese flavoring *Penicillium roqueforti*), alcoholic fermentation, and the conversion of sugars in bread dough to carbon dioxide (*Saccharomyces cerevisiae*). In Asia, *Aspergillus oryzae* is an essential ingredient for the production of soy sauce and the fermented drink, sake. *Rhizopus* spp. secrete a wide variety of enzymes including cellulolytic, proteolytic, lipolytic, and pectinolytic enzymes that are used in the production of various foods such as Tempe from Indonesia [46]. *Rhizopus oryzae* has also been identified as a biocatalyst for biodiesel fuel production [47]. Other fungi such as *Yarrowia lipolytica* have more recent applications in the biodegradation of industrial products [48]. Advances in industrial enzymology following World War II have enabled researchers to identify and utilize various enzymes and proteases that fungi produce to break down carbohydrate and lignin containing plant material in the environment [49]. To date, close to 200 fungal enzymes have been purified from fungal cultures and the biochemical and catalytic properties characterized [10, 50]. These enzymes have great utility in pharmaceutical, agricultural, food, paper, detergent, textile, waste treatment, and the petroleum industries. 

Industrial fungal enzymes are high-molecular-weight proteins that are catalysts [10, 49]. A description of the common enzymes used in various industries is presented in Table 1. The most widely used enzymes of occupational importance are derived from the genus *Aspergillus* and include *α*-amylase, xylanase, and cellulase. A summary of the proteomic and immunologic properties of these enzymes is presented in Table 2. Other enzymes are also utilized from rhizosphere fungal species belonging to the genera *Rhizopus* and *Humicola* (Table 2). These enzymes usually have intracellular or other functional roles associated with apical hyphal growth. It is uncommon for individuals in the general population to be exposed and sensitized to these antigens. In fact, in the general population, the prevalence of sensitization to fungal enzymes has been reported to be as low as 1% and as high as 15% [22, 51]. However, in the occupational environment, workers that handle purified fungal enzymes are at an increased risk of becoming sensitized to enzymes [10, 23, 24, 42, 52–56]. This is especially the case for workers whose occupation requires debagging, sieving, weighing, dispensing, and mixing enzymes [24, 53–56]. Eight-hour time-weighted average exposures demonstrate that occupations weighing the enzyme preparations have the lowest average exposure compared to those workers that sieve [24]. These workers are often exposed to levels of dust that exceed 4 mg m^−3^, the threshold limit value (TLV) for inhalable dust [57]. For other industrial environments that use lipase and cellulose in production, occupational exposure is highest in production areas and laboratories [42].

Adverse health effects associated with enzyme exposure are well characterized in the baking industry. In some countries, bakery exposures to enzymes are one of the leading causes of occupational allergy [58]. Fungal enzymes are commonly used as baking additives to improve the dough, increase shelf life, and decrease production time [19, 49, 59]. Airborne concentrations ranging from 5.3 ng m^−3^ to 200 ng m^−3^ have been reported in occupational environments [12, 59, 60]. Occupational sensitization to fungal enzymes was first reported by Flindt [61]. Later, Baur et al. [62] demonstrated IgE sensitization in workers handling these products. Since the original study, fungal enzymes have been identified as potent allergens in the occupational environment [25, 26]. Prevalence of sensitization to *Aspergillus* enzymes ranges from 8% for glucoamylase [13], 11% for xylanase [13], 13% for cellulase [13], and up to 34% for *α*-amylase [19, 27]. Sensitization to *α*-amylase in bakery workers results in decreased peak expiratory flow [63–66] and OA [20, 28, 67]. In one report, workers exposed to fungal enzymes induced an immediate bronchospastic reaction [49]. In the United States, the prevalence of work-related wheeze, runny nose, frequent sneezing, and specific IgE to fungal enzymes was significantly higher among highly exposed workers [68]. However, other irritant-induced mechanisms associated with high total dust levels have also been reported in a cohort of British bakers [29, 56]. To date, atopy has been hypothesized to be an important risk factor for OA to fungal enzymes.

Occupational exposure to enzymes has been demonstrated in other industries including manufacturing [41, 53, 69], pharmaceutical [25, 38], food processing [70], animal feed, and biotechnology [43]. Like in baking environments, workers handling or in direct contact with fungal enzymes and with a history of atopy are at increased risk of becoming sensitized [10, 23, 24, 42, 52–56]. Sensitization to proteolytic enzymes has also been demonstrated in the manufacture of detergents [53, 71]. In the future, additional uses for fungal enzymes in industrial environments will be identified. Recent examples include the use of *α*-amylase and glucoamylase for the production of ethanol in the biofuel industry [72, 73]. If proper methods of exposure prevention are not followed and exposure is not monitored in these industries, it is possible that new groups of workers will suffer adverse health outcomes and become sensitized to enzymes. In the following sections we describe the major fungal enzymes, prevalence of sensitization, and occupational environment that they are most likely to be encountered.

## 3. Fungal Enzyme Allergens

### 3.1. *α*-Amylase

Fungal amylase is the most well-characterized fungal enzyme used in the occupational environment. Originally discovered by Takamine in 1884 [49], bakers have used *α*-amylase as a supplement to cereal flour to improve carbohydrate fermentation by yeasts and ultimately the quality of the bread [49]. *α*-amylase cleaves long-chain carbohydrates into simpler sugars including maltose [49]. Derived from *A. oryzae*, *α*-amylase is a 478 amino acid glycoprotein with a molecular weight of 53 kDa (Table 2; [6]). Occupational sensitization to *α*-amylase was first reported by Flindt [61] and has subsequently been identified as an allergen in baking [74], pharmaceutical [25], animal feed [12], and biotechnology industries [43]. The allergen was originally designated Asp o 2 by the International Union of Immunological Societies (IUIS) Allergen Nomenclature Subcommittee but now has been redesignated Asp o 21 [27]. Since this preliminary work, *α*-amylase has been identified as one of the principle sensitizers in large-scale bakeries [24, 56]. The prevalence of sensitization among bakers is variable and ranges from 0.9% to 34% [13, 18, 19, 21, 23, 24, 27–35, 54, 66, 67, 75]. Concentrations as high as 40 ng m^−3^ have been reported in baking environments [60]; however, *α*-amylase concentrations in the low ng m^−3^ range have been associated with an increased frequency of sensitization [58].

The most common tasks associated with *α*-amylase exposure involve dispensing, sieving, weighing and mixing [55, 56, 60]. Exposures that exceed the maximum exposure limit for flour dust in the United Kingdom were identified in mixing, weighing [54], and dispensing operations [55]. The prevalence of sensitization to *α*-amylase is 9.9 times greater among workers in high-exposure categories compared to those workers in low-exposure categories [55]. Aerosolized particle size distribution analysis in baking environments demonstrated that workers are exposed to *α*-amylase particles within the inhalable size fraction [60]. OA is commonly identified in workers sensitized to *α*-amylase. After bronchial provocation with *α*-amylase, between 16 and 100% of sensitized workers were found to give a positive immediate response depending on the study [25, 35, 49]. Nasal provocation with *α*-amylase in skin prick test (SPT) positive workers also induced rhinitis [26]. Furthermore, positive associations between *α*-amylase SPT and work-related respiratory symptoms have been identified [23]. Interestingly, heating *α*-amylase has been shown to reduce enzymatic and allergenic activity of the enzyme [76]. Potential sensitization of bakers' family members due to *α*-amylase associated with clothes, shoes, and bakery textiles has also been reported by Vissers [77].

### 3.2. *γ*-Amylase

*γ*-amylase or glucoamylase is primarily obtained from *A. niger, A. awamori, and R. delemar. *Glucoamylase is used as a dough additive by bakers, often in association with *α*-amylase. The enzyme is also used in the production of high-glucose syrups [46]. Glucoamylase has a molecular weight of 68 kDa (Table 2) and can remain functionally active at elevated pH. Glucoamylase exposure has been primarily reported in baking occupations [10, 13, 18]; however, occupational exposure has also been reported in fruit and salad processing [52]. Sen et al. [52] demonstrated that three workers with shortness of breath, chest tightness, and wheeze had specific IgE to glucoamylase. Quirce et al. [18] also demonstrated positive SPT to glucoamylase in all tested subjects (*n* = 4); however, only three of the four patients elicited an early asthmatic response following bronchial provocation. Airborne glucoamylase was shown in 9% of air samples from a bakery [59], and median levels were 10.3 ng m^−3^. Moderate allergenic cross-reactivity has also been reported between glucoamylase and *α*-amylase [18].

### 3.3. Cellulase, Xylanase, and Hemicellulase

Cellulases are enzymes that hydrolize cellulose into glucose and are primarily used in the pharmaceutical, baking, detergent, and textile industries [6, 78]. Cellulase has been purified from several rhizosphere fungi including *A. niger* and *Trichoderma viride* [49], as well as *Humicola insolens* [41]. The molecular weight of cellulases ranges from 22 to 45 kDa (Table 2). Cellulases derived from these fungi are used in baking to break up roughage in dough and as a digestive aid in the food industry [13, 79, 80]. The first case of OA caused by cellulase was reported in 1981 in a plant pathologist [49, 81], and later these findings were confirmed in two pharmaceutical workers [80], four laboratory workers [14], and two bakers [49]. In each of these studies, the workers had specific IgE to the cellulase antigens. In 171 German bakers, the prevalence of sensitization to cellulase was 13% [13]. Airborne concentrations of cellulase have been quantified using a modified dot blot technique and were <180 ng m^−3^ in a flour mill, crisp bread factory, and a bakery [82]. OA has also been reported to cellulase in the baking industry [83] as well as from *H. insolens* used in the detergent industry [41]. 

Endo 1, 4-beta-D-xylanase and beta-xylosidase are major enzymes involved in xylan hydrolysis [13]. Collectively termed xylanases, these enzymes are a type of hemicellulase that breaks down hemicelluloses, a major component in plant cell walls [13]. Besides *α*-amylase, xylanases are the next most frequently used enzymes in the baking industry to remove pentosans from bread and increase bread volume [13, 49]. The prevalence of IgE sensitization to hemicellulase was reported to be 8% [19] and 11% for xylanase [13]. Sander and colleagues [13] found that 7 of 8 bakers had serum IgE to a 105 kDa protein in a xylanase ingredient. This protein was identified using mass spectrometry to be beta-xylosidase derived from *A. niger*. The allergen was designated Asp n 14 by the IUIS Allergen Nomenclature Subcommittee (Table 2). Airborne concentrations of xylanase have been reported to be <40 ng m^−3^ in a flourmill and crisp bread factory [82]. Concentrations as high as 200 ng m^−3^ were also reported in a bakery, but these values were associated with the natural xylanase activity of wheat [82]. Case reports have verified xylanase sensitization and the presence of an IgE mechanism in respiratory disease [15, 79]. OA has also been reported to xylanase in the baking industry [83], and in a case report, a baker had an immediate asthmatic response following inhalation challenge [15]. Cross-reactivity between cellulase and xylanase has been reported to be in the range of 80–90% but no cross-reactivity has been shown with *α*-amylase [13, 14]. Similarly, workers can also be monosensitized to cellulase and xylanase without concomitant sensitization to *α*-amylase [83].

### 3.4. Lactase

*A. oryzae* lactase is a high-molecular-weight protein that is involved in the hydrolysis of the disaccharide, lactose. Lactase is used in the pharmaceutical industry to develop dietary aids for patients intolerant to lactose. In a cross-sectional study of United States pharmaceutical workers, Bernstein and colleagues [38] identified 29% of lactase-exposed workers to have positive SPT response to lactase. Workers with a positive SPT were nine times more likely to have respiratory symptoms than workers with a negative SPT [38]. Interestingly, atopy was not associated with the development of respiratory symptoms. Occupational sensitization to lactase has been reported in workers formulating and packaging gastrointestinal consumer products [39]. In inhalational challenge studies conducted by Laukkanin and colleagues [40], lactase was identified to induce occupational IgE-mediated respiratory sensitization. Interestingly, lactase exposure has also been identified to cause contact skin reactions [40].

### 3.5. Lipase

Lipase is an essential catalyst that digests water-insoluble lipids. *A. oryzae* and *R. oryzae* lipase are used because of low extraction costs, thermal and pH stability, substrate specificity, and activity in organic solvents. Lipase is predominantly used in the manufacture of laundry detergents and in baking; however, other newer applications have been developed. For example, *Candida antarctica* lipase has recently been used as a biocatalyst for the biofuel industry [84]. The incidence of occupational sensitization to lipase, in industrial settings is understudied. In a preliminary analysis of detergent manufacturing workers, 3 workers were found to be sensitized to lipase and bronchial provocation tests provoked a reproducible asthmatic response [41]. A recent case study of a pharmaceutical manufacturing worker also demonstrated sensitization to fungal lipase derived from *R. oryzae* but not *A. oryzaeα*-amylase [44].

### 3.6. Phytase

*A. niger* and *R. oligosporus* produce phosphatase that catalyzes the hydrolysis of phytate to lower-order phosphate esters [16]. Termed phytase, this enzyme enhances phosphate bioavailability in the digestive tract and has been utilized in the animal feed industry during the last two decades [17]. Phytase accounts for 0.1–1% of total extractable protein from *A. niger* [17]. 3-phytase B derived from *A. niger* is an 84 kDa protein that has been designated Asp n 25 by the IUIS Allergen Nomenclature Subcommittee (Table 2). Allergic sensitization to phytase has been reported in animal feed factory workers (7–90%), and sensitization is highest at sites where phytase is handled in powdered form [16, 17, 69, 85]. In a cross-sectional study of 53 technical center workers that produced *A. niger* phytase, 52% of workers in the high-exposure group and only 10% in the low-exposure group were sensitized to phytase [16]. Personal exposure to phytase has been shown to exacerbate OA, and inhalation challenge tests produced immediate asthmatic response [86]. It has been proposed that phytase is highly sensitizing and that direct contact should be avoided in this industry [16].

### 3.7. Enzymes Used in Health Care Settings: Biodiastase and Flaviastase

Fungal enzymes have a number of applications in the healthcare environment. Fungal enzymes derived from *A. niger* are used in powdered form with other enzyme extracts by pharmacists to prepare digestive powders. Biodiastase and Flaviastase are two examples of fungal enzymes that have been associated with sensitization in hospital workers and pharmaceutical workers handling these products [10, 12–21, 23–35, 38–44, 51–88]. To date, health effects in workers exposed to these enzymes remain poorly characterized.

### 3.8. EPg22 Protease: Aspartic Protease

The aspartic proteases produced by *Rhizomucor miehei* and *Cryphonectria parasitica* are utilized in almost half of the cheese production operations throughout the world [46]. The proteases assist in milk clotting and facilitate a change in cheese properties by hydrolyzing certain peptide bonds. Occupational exposure to these proteases has been associated with occupational sensitization in a rennet production plant [11]. Specifically 29% and 6% of workers had a positive skin prick test (SPT) to *R. miehei* and *C. parasitica* aspartic protease extracts, respectively [11]. Other novel enzymes with potential application in the food processing industry have been identified. Pg222 is a novel extracellular protease produced by *P. chrysogenum* (Pg222). The enzyme was isolated from dry-cured hams and was found to have a broad range of applications in industries that produce dry-cured meat products [89]. Although no occupational sensitization has been reported to this enzyme, it demonstrates that the introduction of any new enzyme could potentially represent an occupational hazard.

## 4. Emerging Occupational Fungal Enzyme Exposures

The utility of fungal enzymes to degrade xenobiotics and organic compounds in the industrial sector continues to be recognized [46]. Fungal enzymes are now being used for a variety of purposes across many different industries. Improved biochemical and molecular technologies have enabled the production of other potentially allergenic proteins [14]. According to Baur [10], more than 186 commercial enzymes were produced in the European Union in 2001, and many of these were produced by recombinant technology or had been genetically engineered. Table 1 provides a summary of the major fungal enzymes that are utilized in industrial settings. All of the aforementioned enzymes that are listed in Table 1 have been identified to be potent allergens in the workplace; however, the ability of the other listed enzymes to cause adverse health outcomes following occupational exposure remains unclear. 

Several of the enzymes presented in Table 1, not identified as occupational allergens, have been identified as allergens associated with environmental bioaerosols. Catalase, a fungal enzyme utilized in hygiene products, pharmaceuticals, and textiles, has been identified as an allergen in the entomopathogenic fungus, *Metarhizium anisopliae* [90]. Pectinase is used in brewing and wine production, food processing, and paper industries and allergy to pectinase has been associated with occupational exposure [91]. Esterase has been identified as an allergen in *Hevea brasiliensis* (natural rubber latex) [92]. Beta-glucanase is used to improve the nutritional yield of animal feeds, and occupational exposure has been shown in a case study to significantly reduce forced vital capacity and forced expired volume in 1 second (FEV1) [86]. The worker in this case study was also SPT positive and had specific IgE to beta-glucanase [86]. In the biotechnology and pharmaceutical industries, glutathione-S-transferase (GST) has a number of applications. GST is an approximately 26 kDa protein that has been identified as a major *Alternaria alternata* allergen and is highly conserved across fungi [45, 93, 94]. The IUIS Allergen Nomenclature Subcommittee has designated this allergen Alt a 13 [93, 94]. Interestingly, alpha-galactosidase has been associated with delayed anaphylaxis, angioedema, or urticaria in sensitized patients following the ingestion of beef, pork, or lamb [95]. Although the role of alpha-galactosidase and these other enzymes following occupational exposure remains unclear, these studies provide preliminary insight into the possible potency of these allergens in industrial environments.

## 5. Immunodiagnostic Detection Methodologies

Occupational allergic sensitization to fungal enzymes is diagnosed clinically using available *in vivo* SPT reagents, or *in vitro* assays such as Phadia ImmunoCap [7]. However, SPT reagents for most of the fungal enzymes used in industrial settings are not commercially available and have to be either custom ordered or prepared individually by the investigator. Methods for SPT extract preparation that are used by investigators in the field have been previously described by Quirce et al. [49]. *In vitro* diagnostic tools that can quantify the amount of specific IgE to an occupational allergen are not readily available except in research laboratories where investigators prepare their own inhibition or radioallergosorbent enzyme-linked immunosorbent assay (ELISA) to quantify specific IgE [36, 49]. To date, *α*-amylase (k87) is the only fungal enzyme available on the Phadia ImmunoCap testing panel. To confirm OA caused by fungal enzymes, bronchial provocation tests can be undertaken to document immediate or late-phase responses to fungal enzymes [36, 49]. Positive immediate response criteria used in workers exposed to enzymes include a greater than 20% fall in FEV1, whereas a late-phase response has been considered positive when there is a 30% or greater fall in peak expiratory flow rate [49]. However, there are several limitations with bronchial provocation tests that should be considered; these are discussed in detail by Peden and Reed [7].

In order to better understand the relationships between occupational fungal enzyme exposure and clinical symptomology, accurate information on the distribution and quantity of the fungal enzyme in the occupational environment will be required. Immunodiagnostic methods that utilize antibodies could provide standardized methods for quantifying fungal enzyme biomarkers in a variety of occupational environments. Following validation and interlaboratory comparison, the assays could be used for exposure assessment to determine the existence of exposure-response relationships [58, 96]. This information is critical for the development of future threshold limit values (TLVs) and other occupational standards. 

Several antibodies and immunodiagnostic methods have been produced to detect industrial fungal enzymes, in particular *α*-amylase. These methods have been employed in field investigations and used to quantify the concentration of the enzyme from collected air samples. Bogdanovic et al. [97] used an enzyme immunoassay with a sensitivity of 25 pg/mL to quantify *α*-amylase in airborne and surface dust samples collected from five bakeries. In the same study, a lateral flow immunoassay for *α*-amylase was compared to the reference enzyme immunoassay. The sensitivity of the lateral flow assay was 1–10 ng/mL, and extracts with >5 ng/mL allergen were positive in the lateral flow assay [97]. In a study of 507 personal air samples, Houba and colleagues [60] used a rabbit IgG capture immunoassay to quantify *α*-amylase in specific baking job category. Concentrations of *α*-amylase up to 40 ng m^−3^ were quantified, and workers directly involved with dough preparation had the highest exposures [60]. Using the same rabbit IgG sandwich assay, Nieuwenhuijsen et al. [55] identified dispensing and mixing areas to have the highest *α*-amylase exposure in British bakeries and flour mills. Two monoclonal antibody- (mAb-) based ELISAs have been developed for the detection of *α*-amylase in the occupational environment. Assay sensitivities ranged from 0.2 ng/mL [98] to 0.6 ng/mL [99]. A quantitative mAb-mediated dot blot assay has also been previously described for cellulase and xylase; the detection limits reported were 20 ng m^−3^ and 2 ng m^−3^, respectively [82]. mAbs to other fungal enzymes, such as xylanase have been produced and reported in the literature [100]. Similarly, the detergent industry has produced antibodies and immunoassays for several common fungal enzymes and these have been utilized in industrial hygiene safety programs to mitigate worker exposures [101–103]. Unfortunately, for many other fungal enzymes presented in Table 1, there are no commercially available antibodies to enable quantification in the occupational environment. The development of fungal enzyme-specific mAbs in combination with immunodiagnostic techniques will further our knowledge of the exposure-response relationships in occupational environments. Using these methods will also help enable the development of standards and focus on the prevention of sensitization in heavily contaminated work environments.

## 6. Allergen Avoidance and Directions for the Future

Exposure to fungal enzymes, in particular *α*-amylase, is a considerable health risk in a number of industries. Cross-sectional studies have shown that processing workers in high-exposure categories who handled fungal enzymes are up to ten times more likely to be sensitized to fungal enzymes than workers in the low-exposure category [55]. Highest concentrations of enzymes in the inhalable fraction were encountered among workers located in dispensing, mixing, weighing, and sieving occupations [54–56, 60]. Airborne concentrations as high as 40 ng m^−3^ and in some cases even higher (200 ng m^−3^) have been reported for sensitized workers located in these handling areas [12, 55, 60]. Concentrations in the low ng m^−3^ range have been associated with an increased frequency of sensitization [58]. For other fungal enzymes, such as phytase, similar findings have been reported [16]. 

The continued utilization of other previously overlooked enzymes as well as new genetically engineered enzymes in various industries will continue to provide diagnostic challenges, even for the most seasoned occupational medicine professional. It is likely that new cases of occupational allergic disease will emerge following exposure to fungal industrial enzymes during the next decade. In response, identification of exposure-response relationships will be critical for the development of TLVs and occupational exposure levels. However, this will depend on the development of suitable diagnostic antibodies and immunoassays. Currently, subtilisin, a sereine endopeptidase derived from *Bacillus subtilis*, is the only enzyme for which the American Conference of Governmental Industrial Hygienists (ACGIH) has established a TLV value (60 ng m^−3^). The European Union Directive also classifies the fungal enzymes cellulase and *α*-amylase with the risk phrase R42 (may cause sensitization by inhalation) [10]. There are currently no consensus standards for other industrially utilized fungal enzymes.

As a precautionary measure, it has been concluded that all enzymes should be regarded as an allergen that can exacerbate respiratory sensitization in susceptible populations [10, 59]. Baur [10] has further proposed that all enzymes should be classified as R42 according to the European Union Directive criteria. Although intervention in the bakery industry has had little to no effect [104], installation of engineering controls and implementation of personal protective equipment programs in animal feed workers exposed to phytase was shown to result in the immediate cessation of hypersensitivity symptoms [10]. Improvements in biotechnology have also included the encapsulation of some enzymes [105, 106] and proteins [107]. These engineering controls have been proposed to reduce occupational exposure to enzymes; however, encapsulation alone may not completely prevent enzyme-induced allergy and OA [108, 109]. To date, the detergent industry has implemented a derived minimal effect level (DMEL) of 60 ng m^−3^ for pure enzyme proteins [110]. Although this DMEL was provided as guidance by the ACGIH, other manufacturers have implemented their own occupational exposure guidelines (OEGs) for fungal enzymes such as *α*-amylase (5–15 ng m^−3^), lipase (5–20 ng m^−3^), and cellulase (8–20 ng m^−3^) [110]. In addition, the detergent industry has developed a medical surveillance program to identify and correct elevated exposures before occupational illnesses occur [101–103, 111]. As a result, the incidence of occupational allergy has dropped substantially [101–103]. Implementation of DMELs and OEGs will further assist in the reduction of occupational exposure. Reducing worker exposure to fungal enzymes in industry by the implementation of engineering controls and other allergen avoidance strategies will continue to mitigate personal exposure and further reduce the occupational health risk.

## Figures and Tables

**Table 1:** Fungal enzymes utilized in different industries and associations with occupational sensitization.

Industry	Fungal Enzyme	
	Characterized occupational allergen	Uncharacterized occupational allergen
Agriculture	Protease, lipase	
Animal feed	*α*-amylase, cellulase, lipase, phytase, protease, and xylanase	Beta-glucanase*, endo-xylanase
Pulp and paper production	Cellulase, hemicellulase, lipase, and xylanase	Esterase*, laccase, lignin peroxidase, manganese peroxidase, pectinase*, and mannose
Waste management	Lipase	Esterase*, cytochrome P450, laccase, lignin peroxidase, manganese peroxidase, and monooxygenase
Biotechnology	*α*-amylase, cellulase, glucoamylase, hemicellulase, and protease	Cytochrome P-450 monooxygenase, glucose oxidase, glutathione-transferase*, lignin peroxidase, and manganese peroxidase,
Detergent	*α*-amylase, cellulase, lipase, and protease	
Food processing	*α*-amylase, cellulase, glucoamylase, lactase, lipase, protease, and xylanase	Glucose isomerase, invertase, and pectinase*
Biofuels	*α*-amylase, cellulase, glucoamylase, protease, and xylanase	
Bakery	*α*-amylase, cellulase, glucoamylase, hemicellulase, lipase, protease, and xylanase	Glucose oxidase, lipoxygenase
Brewing and wine production	Cellulase, glucosidase, protease, and xylanase	Alpha-acetolactate decarboxylase, beta-glucanase*, and pectinase*
Pharmaceutical	Lactase, lipase, and protease	Alpha-galactosidase*, catalase*, cytochrome P450 oxygenase, and glutathione transferase*
Textile	*α*-amylase, cellulase, lipase, protease, and xylanase	Catalase*
Leather processing	Lipase, protease	
Hygiene products	Glucoamylase, protease	Catalase, *glucose oxidase

Adapted from Baur [10] and from the Concordia University Genomics Project website: https://fungalgenomics.concordia.ca/home/indappl.php.

*Denotes fungal enzymes associated with allergic sensitization in other fungal bioaerosols or environmental allergens.

**Table 2:** Fungal enzymes associated with occupational sensitization and asthma in occupational environments.

Fungal Order	Fungal species	Allergen	Putative function	Calculated size (kDa)	Accession number	Patient reactivity	Occupational environment	Reference
Ascomycota								
Diaporthales	*Cryphonectria parasitica*	Cry p AP	Aspartic protease	43	X63351	5.7%^†^	Food processing	[11]
Eurotiales	*Aspergillus niger*	Asp n 14*	Beta-xylosidase	105	AF108944	4–11%^††^	Bakers, textile, detergent, animal feed	[12–15]
		Asp n 25*	3-phytase B (phosphatase)	84	L20567	37%^††^	Animal feed	[16, 17]
		Asp n glucoamylase	Glucoamylase	68	X00548, AM270061	5–19%^†^ 63%^††^	Bakers	[10, 13, 18]
		Asp n hemicellulase	Hemicellulase	22.6	A19535	8–43%^†^ 100^††^	Bakers	[19–21]
	*Aspergillus oryzae*	Asp o 21*	TAKA-amylase A	53	X12725, X12727, M33218, D00434	0.9–35%^†^ 1–32%^††^	Bakers, pharmaceutical	[12, 20–37]
		Asp o lactase	Lactase		AP007164	29%–31^†^	Pharmaceutical	[38–40]
		Asp o lipase	Lipase			SR	Detergent	[41]
	*Thermomyces lanuginosus (Humicola lanuginosa)*	The l lipase	Lipase	32	EU370914, AF054513	3%^††^	Detergent, food processing	[42]
Hypocreales	*Trichoderma viride*	Tri v cellulase	Cellulase	42	EF602036	35%^††^		
	*Trichoderma reesei*	Tri rs cellulase	Cellulase	48	AY928809	13%^†^	Biotechnology	[43]
Sordariales	*Humicola insolens*	Hum in cellulase	Cellulase	45	P56680	SR	Detergent	[41]
Zygomycota								
Mucorales	*Rhizomucor miehei*	Rhi m AP	Aspartic protease	46	M18411	28.6%^†^	Food processing	[11]
	*Rhizopus oryzae*	Rhi o lipase	Lipase	42	M38352, AB433531, AF229435	SR	Pharmaceutical	[44]

Adapted from the IUIS Allergen Nomenclature Subcommittee, Allergome (http://www.allergome.org/), and [45].

*Denotes allergens deposited to the IUIS Allergen Nomenclature Subcommittee.

SR: Patients with a positive SPT or specific IgE.

—^†^Denotes that patients were tested with SPT.

—^††^Denotes that patients were serologically screened using Pharmacia UniCap, Rast, or Immunoblot.
